# *Absidia Corymbifera *in an immune competent accident victim with multiple abdominal injuries: case report

**DOI:** 10.1186/1471-2334-7-46

**Published:** 2007-05-25

**Authors:** Rita Belfiori, Adelmo Terenzi, Laura Marchesini, Antonella Repetto

**Affiliations:** 1 Intensive Care Unit, Perugia General Hospital, Monteluce, Perugia, Italy; 2 Hematology and Clinical immunology Section. Dept. of Clinical and Experimental Medicine, University of Perugia, Italy; 3 Microbiology Unit, Dept of Experimental Medicine and Biochemical Sciences, University of Perugia, Italy

## Abstract

**Background:**

We report a case of mucormycosis in a healthy 17-year-old accident victim with multiple abdominal injuries which was caused by infection with *Absidia Corymbifera*, a ubiquitous saphrophyte in the ground.

**Case presentation:**

The patient was admitted to hospital with massive abdominal trauma. During an 8-hour emergency operation he received transfusions of compacted red blood cells, plasma, platelets and hemagel. He developed a crush syndrome with acute renal failure, resolved with extra-corporeal dialysis and had to undergo splenectomy because of spleen hematoma. As wound secretion and central venous catheter (CVC) blood cultures and drainage fluid were positive for *Enterococcus Faecium*, *Providentia Rettgeri*, *Hafnia Alvei *and *Candida Albicans*, tecoplanin, metronidazole, imipenem, and flucanozole were administered.

Although the CVC was changed high fever persisted and discharge continued from the large abdominal wound. Repeated tampons in different sections and wound secretion smears were positive for *A. corymbifera*. Flucanozole was stopped and liposomal amphotericin (Ambisome; 5 mg/Kg i.v.) given for over 3 months.

The patient improved; fever gradually disappeared. After 8 days, tampons and wound secretion smears were negative for *A. corymbifera*. No other fungal infections developed. Drainage fluid was later positive for tecoplanin-resistant *E. faecium *and *Pseudomonas Aeroginosa *responding only *to *meropenem and ciprofloxacin. Abdominal computerized tomography visualized fluid accumulation around the iliac-femoral bypass. Abcess was ruled out when scintigraphy showed no tracer uptake. The lesion was drained. Drainage fluid cultures were negative for bacteria and fungi. Fluid accumulation gradually disappeared with prolonged antibiotic and antifungal therapy.

One year after the accident the patient is in good health, with normal quality of life.

**Conclusion:**

Successful outcome was due to early, specific antifungal therapy, at sufficiently high dosage which was prolonged for an adequate period of time. Early diagnosis of mucormycosis is essential for efficacious anti-fungal treatment and prevention of irreversible spread of mucormycosis to vital organs. It presupposes awareness that *A. corymbifera *infection can develop in healthy individuals who are stressed and traumatized through skin-ground contact in accidents.

## Background

Absidia genus, a fungus belonging to the Mucorales group of the Zygomycetes class, is an ubiquitous saphrophyte in the ground which may cause zygomycosis. Although those infections have occasionally been reported in immune competent patients after accidents or trauma involving contamination through skin-ground contact [1-3], they usually occur in immune depressed hosts e.g. patients undergoing chemotherapy for cancer or leukaemia, patients with poorly controlled diabetes, particularly if ketoacidosis is present, those with iron accumulation, those undergoing chelant therapy, haemodialysis or prolonged steroid therapy, patients who are positive for human immunodeficiency virus (HIV) or who have suffered severe, extensive burns [4-6]. Symptoms range from a skin infection around wounds to systemic multi-visceral involvement. Zygomycetes infection is confirmed by hyphae in tampon cultures that are refractory to antibiotic therapy and by wound secretion positivity at microscopy. Histology is not always positive for Absidia Corymbifera [4]. Here we report a case of mucormycosis in an apparently immune competent accident victim with multiple abdominal injuries.

## Case presentation

A healthy 17-year-old Caucasian male weighing 70 Kilograms was infected with *Absidia Corymbifera *after suffering major abdominal injuries in an accident with agricultural machinery. The patient was admitted to hospital with multiple abdominal muscle lacerations and soil contamination of the abdominal wall, haemorrhage from the right internal iliac artery, multiple vein lacerations, contusions and obstruction of the right external iliac artery at the iliac-femoral junction, superficial femoral vein laceration, ruptured intestine and colon, fractured pelvis with bone detachment, right retroperitoneal hematoma and haemorrhagic shock.

In an 8-hour emergency operation surgeons constructed an external iliac-right common femoral artery bypass, inserted ligature of the right hypogastric artery, sutured iliac-femoral vein lacerations, resectioned the small intestine and left colon and sutured lacerations in the rectum, right abdomen and psoas. During surgery the patient received transfusions of 11 units of compacted red blood cells, 9 units of plasma, 10 units of platelets and 9 litres of hemagel.

After surgery the patient was admitted to the Intensive Care Unit (ICU) because of marked hypothermia and low blood pressure. Blood tests showed: white blood cells 10 × 10^9^/L; haemoglobin12.4 g/dl; platelets: 124 × 10^9^/L; prothrombin: 55%; partial thromboplastin time: 39.7 sec; International Normalized Ratio = 2; fibrinogen 195 mg/dl. Blood chemistry indicated nitrogen 33 mg/dl; creatinine 11 mg/L; creatinine clearance 100 ml/min; proteins 3.9 g/dl; albumin 1.7 g/dl; aspartate aminotransferase 101 UI/L; alamine aminotransferase 36 UI/L; creatine phosphokinase (CPK) 8436 UI/L; lactic dehydrogenase (LDH) 930 UI/L; myoglobin >500 ng/ml; normal electrolyte balance; glucose 90 mg/dl; lactate 43.1 mg/dl (normal range 5.7–22). The patient was HIV negative. As drainage fluid was positive for *Enterococcus Faecium*, *Providentia Rettgeri*, *Hafnia Alvei *and *Candida Albicans*, tecoplanin (6 mg/Kg i.v. once daily), metronidazole (7.5 mg/Kg i.v. daily 4 times daily) imipenem (10 mg/Kg i.v. thrice daily) and flucanozole (8 mg/Kg i.v. once daily) were administered. Twelve hours after admission to the ICU he developed a crush syndrome with acute renal failure requiring immediate extra-corporeal dialysis. Dosages of all drugs were adjusted to renal function.

### Day +3

Severe acute renal failure was partially resolved. No urination; creatinine 57 mg/L; clearance 4.30 ml/min; CPK 95700 IU/L (normal value < 190 UI/L); myoglobulin >500 UI/L; LDH 4733 IU/L. Potassium 7.1 mEq/L. Four days later extracorporeal dialysis was stopped.

### Day +4

The patient underwent splenectomy because of spleen hematoma with peritoneal bleeding. 800 ml blood were removed.

### Day +6

Surgical wound secretion resulted positive for *A corymbifera*, *E. faecium*, *P Rettgeri*, *H. alvei *and *C. albicans*. Wound secretion and CVC blood cultures were positive for *C. albicans*. *A. corymbifera *positivity was considered accidental contamination due to residual soil traces.

### Day + 10

**Figure 1:**
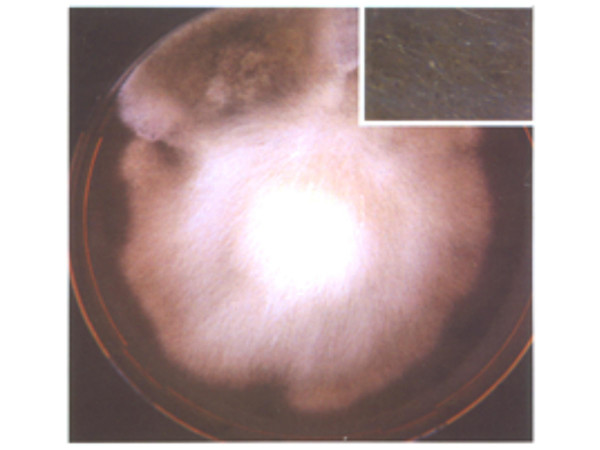
*A. corymbifera *colony in wound secretion culture.

**Figure 2:**
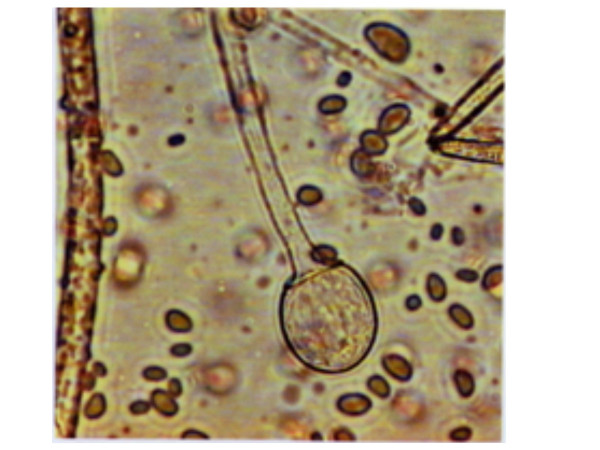
Microscopy (magnification × 100). Abdominal fluid: *A. corymbifera *growth in culture with sporangium and cone-shaped columella.

**Figure 3:**
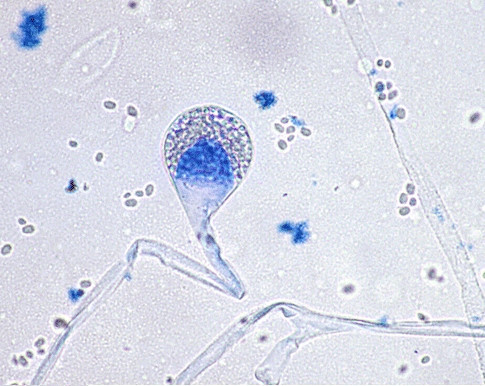
Microscopy (magnification × 100). *A. corymbifera *: Typical broad non-separate hyphae showing right-angled branching.

Although the CVC had been changed very high fever persisted. The patient was affected by vomiting and confusion. Discharge continued from the large, hyperemic, foul-smelling, abdominal wound. Surgeons drained abcess fluid from the right abdominal wall. Cultures of abcess fluid and drainage, as well as wound secretion smears, were positive for *A. corymbifera *(figs 1, 2 and 3). Growth was observed in mycetes medium and confirmed in Saburaud agar where greyish-white, woolly, flock-like colonies were observed 48 hours later. An antimicrogrammme indicated resistance to 5-fluorocytosine, flucanozole and voriconazole and response to amphotericin B with a low antimicrogramme index (0.016 μg/ml). Posaconazole was not tested as it was not available the hospital pharmacy. Flucanozole was suspended and liposomal amphotericin (Ambisome) started (5 mg/Kg i.v. for 40 days). Therapy was well tolerated.

### Day + 15

The patient started to improve and fever gradually dropped.

### Day +18

Tampons and wound secretion smears were negative. Blood cultures for *C. albicans *were also negative. No other fungal infections developed.

### Day +20

Drainage fluid was positive for *E. faecium *and *Pseudomonas Aeroginosa*. As *E. faecium *was resistant to tecoplanin and vancomycin, linezolid (10 mg/Kg i.v. twice daily) and rifampycin (15 mg/Kg i. v. twice daily) were administered. *P. Aeroginosa *responded only to meropenem (13 mg/Kg i.v. thrice daily) and ciprofloxacin (6.5 mg/Kg i.v. thrice daily). This combined antibiotic therapy was continued until discharge from the ICU.

### Day + 35

Abdominal computerized tomography visualized an area of 12 × 5.5 cm fluid accumulation around the iliac-femoral bypass. Abcess was ruled out when scintigraphy of autologous radio-labelled leucocytes showed no tracer uptake.

**Figure 4:**
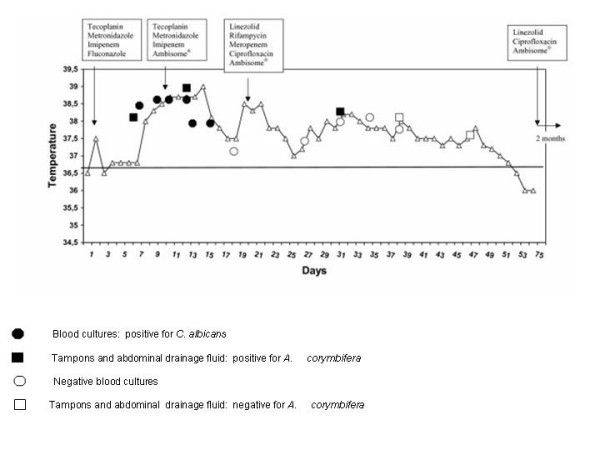
Clinical course and microbiological cultures. • Blood cultures: positive for *C. albicans*. ■ Tampons and abdominal drainage fluid: positive for *A. corymbifera*. ◯ Negative blood cultures. □ Tampons and abdominal drainage fluid: negative for *A. corymbifera*.

Given the risks of further surgery, the relatively complex surgical technique that would be required, the patient's 25% loss of body weight despite parenteral nutrition with 2,500 calories daily and negative scintigraphy, it was decided only to drain the lesion. Drainage fluid cultures were negative for bacteria and fungi, confirming the validity of therapy. Fluid accumulation gradually disappeared with prolonged antibiotic and antifungal therapy. (Figure 4 shows the patient's clinical course). After 75 days in the ICU the patient was transferred to the Infectious Diseases Unit where he continued anti-infectious treatment with liposomal amphotericin B, linezolid and ciprofloxacin for another two months. One year after the accident he is in good health, with normal quality of life.

## Discussion

The massive abdominal trauma could have facilitated abdominal colonization by *A. corymbifera*. Systemic factors – acute renal failure, multiple fractures, soft tissue destruction, haemorrhagic shock, transfusions, antibiotic therapy, parenteral nutrition – undoubtedly stressed the patient's immune status and facilitated fungal growth. Mucor's marked affinity for blood vessels, determining necrosis rather than abcess, probably accounts for our negative scintigraphy. One may hypothesize the fluid was fungal-related and that antifungal therapy and drainage gradually eliminated it. Fever disappeared completely a long time after cultures became negative because, in patients with major abdominal injuries, bacterial abdominal infections require prolonged therapy with specific antibiotics

Cocanour et al., [7] reported 9 cases of skin mucormycosis caused by contact with the ground following trauma. Overall mortality is 30% which rises to 100% in cases of cranial and brainstem trauma because of necrosis in soft tissues adjacent to the brain and the ensuing encephalitis, as is observed in cases of endoophthalmitis [8]. In these 9 patients abdominal involvement was not fatal. On the other hand, Horre et al., [9] reported simultaneous infection with *C. albicans *and *A. corymbifera *in a healthy patient who suffered multiple rib fractures, kidney, liver and pancreas rupture in a road accident. After surgery, the abdomen wall was infected with both fungi and, despite amphotericin B therapy, the patient died of multi-organ failure. Our young patient probably survived similar infections because, apart from the spleen, his vital abdominal organs were injured less severely in the accident.

In *A. corymbifera *infection amphotercin B is more efficacious in vitro and in vivo than voriconazole or itraconazole [10,11]. Posaconazole, which is also efficacious in vivo and in vitro [11], has been successfully administered in cases of amphotericin B intolerance [12], as salvage therapy for zygomycosis [13] and, in combination with amphotericin B, as therapy for disseminated infection [14]. Consequently, treatment of choice for *A. corymbifera *infection is early therapy with amphotocerin B alone or in combination with posaconazole, together with surgical asportation of necrotic tissue to remove the infected area [15]. As our patient had been affected by acute renal failure we opted for liposomal amphotercin B which ensured prolonged treatment at a sufficient dosage and no organ toxicity. We also preferred drainage to any further surgery.

## Conclusion

In this patient with concomitant *A. corymbifera *and *C. albicans *infections, successful outcome was due to early, specific antifungal therapy at sufficiently high dosage which was prolonged for an adequate period of time. Early diagnosis using wound secretion cultures and smears is essential if anti-fungal treatment is to be efficacious and prevent irreversible spread of Zygomycetes to vital organs. It presupposes awareness that *A. corymbifera *infection is not restricted to immune compromised hosts but can develop in healthy individuals who are stressed and traumatized through skin-ground contact in accidents.

## Abbreviations

CVC = Central Venous Catheter

ICU = Intensive Care Unit

CPK = Creatinine Phosphokinase

## Competing interests

The author(s) declare that they have no competing interests.

## Authors' contributions

Rita Belfiori took care of the patient in the ICU and drew up the first draft of the report, Adelmo Terenzi, consultant haematologist, made a substantial contribution to analysis and interpretation of clinical and microbiological data and revised the manuscript critically Laura Marchesini took care of the patient in the ICU and drew up the figures Antonella Repetto carried out all microbiological studies and collected all microbiological data

## Pre-publication history

The pre-publication history for this paper can be accessed here:


